# Impact of a prospective feedback loop on care review activities in older patients at the end of life. A stepped-wedge randomised trial

**DOI:** 10.1186/s12877-022-03554-x

**Published:** 2022-11-16

**Authors:** Christine Brown, Xing J. Lee, Alison Farrington, Carla Shield, Hannah E. Carter, Steven M. McPhail, Magnolia Cardona, Kenneth Hillman, Leonie Callaway, Lindy Willmott, Ben P. White, Gillian Harvey, Nicholas Graves, Adrian G. Barnett

**Affiliations:** 1 Australian Centre for Health Services Innovation and Centre for Healthcare Transformation, School of Public Health and Social Work, Queensland University of Technology, Kelvin Grove, Queensland Australia; 2 Digital Health and Informatics Directorate, Metro South Health, Woolloongabba, Brisbane, Australia; 3 Institute for Evidence Based Healthcare, Bond University, Robina, Queensland Australia; 4 Evidence Based Practice Professorial Unit, Gold Coast University Hospital, Southport, Queensland Australia; 5 Simpson Centre for Health Services Research, South West Sydney Clinical School, University of New South Wales, Liverpool, New South Wales Australia; 6 School of Clinical Medicine, University of New South Wales, Liverpool, New South Wales Australia; 7 Ingham Institute for Applied Medial Research, Liverpool, New South Wales Australia; 8 Royal Brisbane and Women’s Hospital, Herston, Queensland Australia; 9 Faculty of Health, Queensland University of Technology, Kelvin Grove, Queensland Australia; 10 Faculty of Medicine, University of Queensland, Herston, Queensland Australia; 11 Australian Centre for Health Law Research, School of Law, Faculty of Business and Law, Queensland University of Technology, Brisbane, Queensland Australia; 12 College of Nursing and Health Sciences, Flinders University, Bedford Park, South Australia Australia; 13 Duke-NUS Postgraduate Medical School, National University of Singapore, Singapore, Singapore

**Keywords:** Non-beneficial treatment, Prospective feedback loop intervention, Stepped-wedge trial, Advance care planning, Older people, End of life

## Abstract

**Background:**

Hospitalisation rates for older people are increasing, with end-of-life care becoming a more medicalised experience. Innovative approaches are warranted to support early identification of the end-of-life phase, communicate prognosis, provide care consistent with people’s preferences, and improve the use of healthcare resources. The Intervention for Appropriate Care and Treatment (InterACT) trial aimed to increase appropriate care and treatment decisions for older people at the end of life, through implementation of a prospective feedback loop. This paper reports on the care review outcomes.

**Methods:**

A stepped-wedge randomised controlled trial was conducted in three large acute hospitals in Queensland, Australia between May 2020 and June 2021. The trial identified older people nearing the end of life using two validated tools for detecting deterioration and short-term death. Admitting clinical teams were provided with details of patients identified as at-risk with the goal of increasing awareness that end of life was approaching to facilitate appropriate patient centred care and avoid non-beneficial treatment. We examined the time between when the patient was identified as ‘at-risk’ and three outcomes: clinician-led care review discussions, review of care directive measures and palliative care referrals. These were considered useful indicators of appropriate care at the end of life.

**Results:**

In two hospitals there was a reduction in the review of care directive measures during the intervention compared with usual care at 21 days (reduced probability of − 0.08; 95% CI: − 0.12 to − 0.04 and − 0.14; 95% CI: − 0.21 to − 0.06). In one hospital there was a large reduction in clinician-led care review discussions at 21 days during the intervention (reduced probability of − 0.20; 95% CI: − 0.28 to − 0.13). There was little change in palliative care referrals in any hospital, with average probability differences at 21 days of − 0.01, 0.02 and 0.04.

**Discussion:**

The results are disappointing as an intervention designed to improve care of hospitalised older people appeared to have the opposite effect on care review outcomes. The reasons for this may be a combination of the intervention design and health system challenges due to the pandemic that highlight the complexity of providing more appropriate care at the end of life.

**Trial registration:**

Australia New Zealand Clinical Trial Registry, ACTRN12619000675123 (registered 6 May 2019).

**Supplementary Information:**

The online version contains supplementary material available at 10.1186/s12877-022-03554-x.

## Background

The problem of inappropriate and non-beneficial care for older people nearing the end of life is well documented. Studies have shown non-beneficial treatment at the end of life to have an incidence of 12% [1] and a prevalence of 33 to 38% [2]. The reasons why non-beneficial treatments are provided are multifaceted, having been linked to factors arising from clinician, hospital, and patient levels [3–5]. Addressing these factors is challenging, especially in large, complex acute care settings. Despite many attempts at providing more appropriate care for older people, there have been few successful interventions reported in the literature [6]. This likely reflects that scenarios in clinical practice settings near the end of life are not simple, consistent, logical, linear, or predictable. They are complex and uncertain with patients’ preferences influenced by age, physical and cognitive function, culture, family preferences, clinician advice, financial resources, and perceived caregiver burden [6].

Clinicians providing end-of-life care are often tasked with preparing patients and families for a transition to less active treatment [7], however they can frequently experience a range of barriers in providing that care pathway. These barriers may lead to an increase in treatment provided that is not beneficial to the patient [4, 8]. Further, they can cause moral distress to clinicians and increase risk of a bad death by prolonging or increasing patient suffering [9]. There is a risk that non-beneficial treatment can occur due to: failure to put the person at the centre of interventions; the reluctance of the medical profession to discuss end-of-life issues and their lack of education and training in the area; and the expectations of society around medical miracles and the emphasis on what medicine can achieve rather than what it can’t [8].

The aim of the InterACT trial was to increase appropriate care towards the end of life for older patients in hospital settings. Here we present the results for three of the immediate impact measures of clinicians’ response to the intervention using the trial data currently available: clinician-led care review discussion; review of care directive measures; and palliative care referral. Other patient and cost outcomes analyses are outlined in our study protocol [10] and results will be reported once these data are available.

## Methods

### Design

**Fig. 1:**
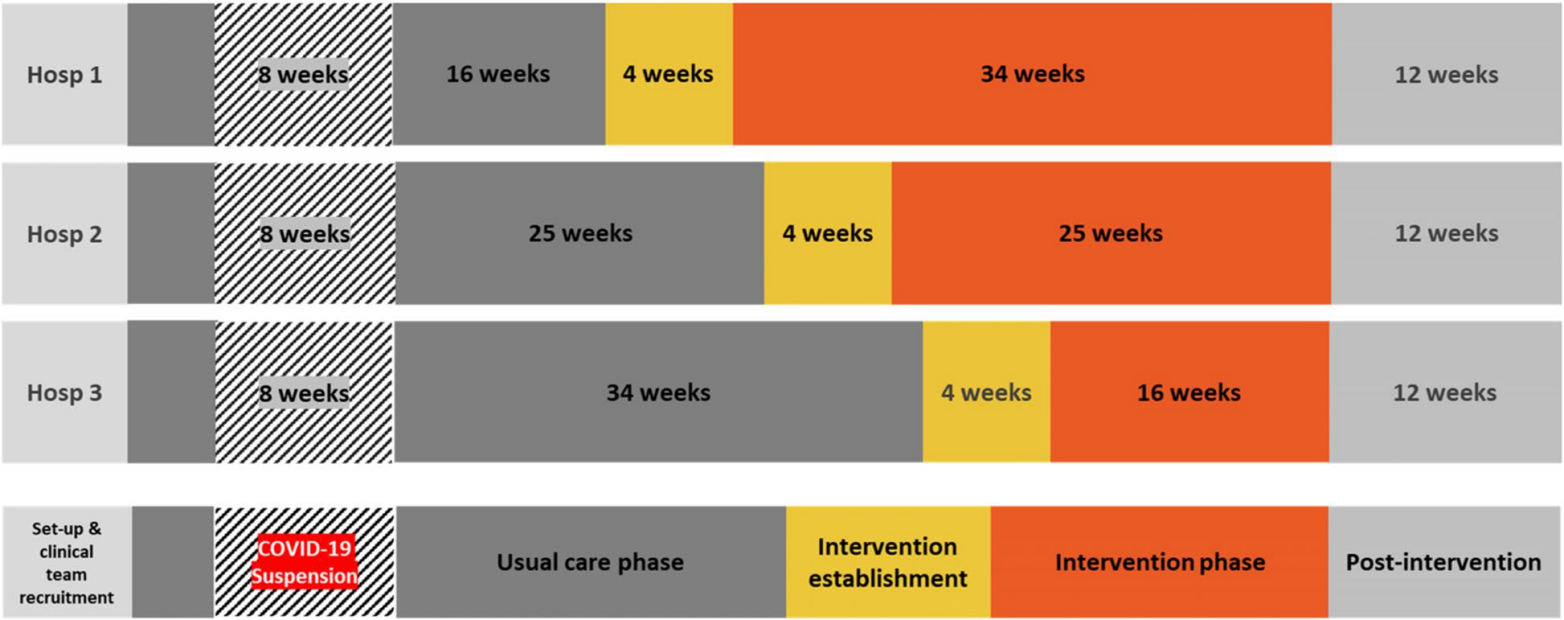
Stepped-wedge design showing the usual care and intervention periods in the three hospitals

The InterACT study was a multi-centre randomised controlled trial using stepped-wedge roll-out with five stages across three large acute hospitals in Queensland, Australia (Fig. 1).

The three participating hospitals were randomly allocated to intervention timing through the allocation of hospital identifiers from 1 to 3 prior to commencement of the trial. These identifiers dictated the allocation to the stepped-wedge design, as per Fig. 1. All hospitals and their participating clinical teams completed the site preparation and clinical team recruitment phase in February 2020 and commenced the usual care phase four weeks later. The usual care phase was disrupted by an eight-week trial suspension due to COVID-19, with the usual care phase restarting in May 2020. The usual care phase was randomised by hospital to be either 16, 25 or 34 weeks, followed by a four-week establishment phase, and an intervention phase of either 16, 25 or 34 weeks until June 2021. The total data collection period was 54 weeks.

### Participants

The three participating hospitals were tertiary level public hospitals in South-East Queensland, Australia. All three hospitals provide an extensive range of clinical services to all ages, and all have palliative care teams. A purposive sampling process identified clinical teams that had a consistent and substantial number of older patient admissions. Following consultation with a local hospital executive advisory group, invitations to participate were emailed to clinical team leads who had participated in a trial information session. Up to seven clinical teams were permitted from each hospital. Fourteen teams consented across the three hospitals, and were a combination of general medicine, cardiology, orthopaedics, renal medicine, thoracic medicine, stroke, neurosurgery, and vascular.

### Patient record screening

Patients were considered eligible for screening if they were 75 years or older and admitted under a clinical team who were enrolled in the trial. Patient admission records were screened on Mondays and Thursdays each week from commencement of the usual care phase until completion of the intervention phase in each hospital (54 weeks). Screening was completed by senior registered nurses, employed by the trial, and trained in the use of the screening tools and the study database. The Criteria for Screening and Triaging to Appropriate aLternative care (CriSTAL) [11] and the general indicators of the Supportive and Palliative Care Indicators Tool (SPICT) [12] were used to screen and determine individual risk of short-term death (“at risk”) (Additional file 1: screening tools).

The patient was deemed at-risk if they had a CriSTAL score of 6 or more or a SPICT score of 2 or more. Two teams in one hospital found that the SPICT tool lacked discrimination, as it was positive for over 95% of their patients, partly because almost every patient was an unplanned hospital admission. After discussion with the wider research team, these two teams increased the SPICT threshold from 2 to 3 as they believed this would more usefully differentiate risk in their patient cohort. This change happened at 203 days (29 weeks) and 304 days (43 weeks) into the trial.

### The intervention

The intervention was a prospective feedback loop notifying clinicians of any patients under their care determined as at-risk. We sought feedback from key stakeholders that was used to refine the intervention design. This included meetings with nurses, clinicians, directors and senior policy staff. We used focus groups with clinicians to get their feedback on the criteria used to signal futile care and how the intervention would be implemented. There was a three-person health consumer group convened with Health Consumers Queensland to get feedback on the acceptability of the intervention to patients and families. A two-fold notification system was used. The first was a real time notification that was different in each hospital - two hospitals used an alert attached to the patient electronic record or medical handover report, and the other hospital used a visual flag displayed on the electronic patient journey board in each ward. The second notification was an email sent to any at-risk patients’ clinician and the end of each screening day. This was a pragmatic trial and during the establishment phase the clinical teams were supported to tailor a clinical response to screening feedback that was feasible and practical to implement throughout the intervention phase.

### Protocol deviation

These results are presented for the hospitals separately, which was a departure from the protocol. This decision was made because the cumulative incidence curves had strikingly different patterns between hospitals in terms of times to discharge and outcome. A combined result is dominated by the largest hospital, and does not reflect the typical patient flow in the other hospitals.

### Outcomes

The three outcomes examined were chosen as they are important indicators recognised to prevent non-beneficial treatments towards the end of life [2]. These three outcomes were: clinician-led care review discussion, review of care directive measures and, palliative care referral. Outcomes were measured from the time each patient was admitted under the participating team to the time of the first documented occurrence of each outcome.

The medical notes of each patient identified as at-risk were reviewed by trained registered nurses for evidence of completion of any of the three outcomes. Medical notes were reviewed each audit day until either: the outcome was completed; the patient was discharged from the team or the hospital; or the two-week follow up period was completed.

Two of the three hospitals used electronic medical records, allowing any non-completed outcomes to be retrospectively checked for each at-risk patient. The third hospital used paper medical records; retrospective screening was only completed on at-risk patients where no outcomes were completed in prospective screening, which accounted for 25% of the at-risk patients at this hospital.

The ‘review of care directives’ outcome, specifically the completion of an acute resuscitation plan, was deemed completed ‘prior’ when there was an existing and valid document (correctly completed less than 12 months ago) in the patient record.

### Statistical methods

We used cumulative probability curves to investigate differences between usual care and the intervention whilst accounting for the competing risks of discharge and death. These plots show the cumulative probability of the outcomes over admission time, and hence illustrate both the timing of outcomes and the overall difference between the usual care and intervention phases. Patients were censored at the end of the trial when data collection ended and they were still in hospital, or if they were still in hospital during the four-week establishment (change-over) from the usual care to intervention phase. To avoid fishing and multiple re-analyses of the data, we first created a complete set of results using a scrambled intervention group. The final results using an unscrambled treatment were not created until all investigators were satisfied with the planned analyses [13].

We estimated the cumulative probability of the outcomes at 21 days, when 98% of patients had been discharged or experienced the outcome and calculated the difference between the usual care and intervention phases. We used a Fine–Gray sub-distribution hazard model that examined the impact of the intervention on the cumulative probabilities over time for patients who had yet to experience the outcome [14].

The large differences in the results between hospitals led us to present the results at a hospital level and not give a combined estimate. See Additional file 2: statistical information, for further details on the statistical methods.

The analysis was made using R version 4.1.1 [15]. All the R code is publicly available on GitHub: https://github.com/agbarnett/InterACT.

## Results

The trial screened 7293 patients, with 4305 (59%) deemed at-risk. Descriptive characteristics of the patients for the usual care and intervention phases indicate an elderly and vulnerable group with a majority at-risk of death (see Table 1). The summary statistics are similar and indicate no clear differences between the usual care and intervention phases.

**Table 1:** Basic characteristics of the patients by intervention phase. Results combined across the three hospitals. Row three shows the percent at-risk, and the following rows are for at-risk patients only

Characteristic	Usual care phase	Intervention phase	Total
Weeks	75	75	150
Patients screened, n	3786	3507	7293
At-risk, n (%)	2160 (57%)	2145 (61%)	4305 (59%)
Female, n (%)	1128 (52%)	1200 (56%)	2328 (54%)
Age, median (IQR)	84 (79 to 88)	84 (79 to 89)	84 (79 to 89)
CriSTAL, median (IQR)	5 (4 to 6)	5 (4 to 6)	5 (4 to 6)
SPICT, median (IQR)	2 (2 to 3)	3 (2 to 3)	3 (2 to 3)

**Fig. 2:**
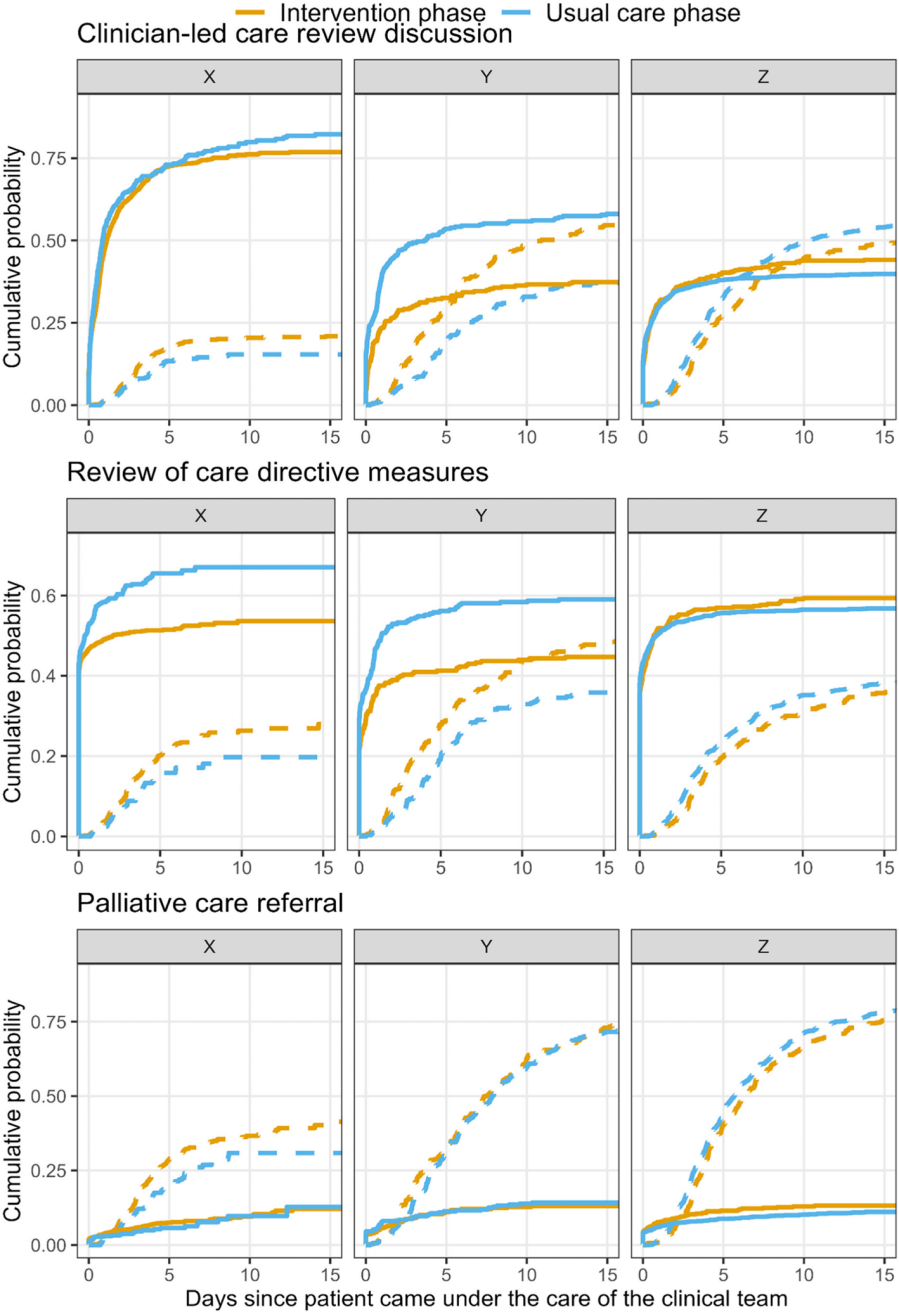
Cumulative probability curves for the three outcomes (solid lines) and the competing risk of discharge/death (dotted lines) by blinded hospital names. The x-axis is the time since the patient came under the care of the clinical team and ends at 15 days, which focuses on most events and illustrates the rapid changes in the first few days of the patients’ admission to the enrolled clinical teams. The y-axis is the cumulative probability of the outcome or death/discharge. The upper limits on the y-axes vary by outcome

The cumulative probability curves are in Fig. 2 with a version including confidence intervals shown in Additional file 3: Fig. S1. The number of outcomes is in Additional file 3: Table S1.

In Hospitals X and Y, patients in the usual care phase were more likely to have experienced a clinical-led review discussion and review of care directive measures than patients in the intervention phase, whereas the curves for Hospital Z show a slight improvement on both outcomes during the intervention phase. The differences between the usual care and intervention phases for palliative care referrals were small in all three hospitals. Discharges were generally faster in the intervention than usual care phase in Hospitals X and Y, which reduced opportunity for audit and may explain why fewer outcomes were observed in the intervention phase. The survival data are illustrated in Additional file 3: Fig. S2.

The results from the probability curves during the intervention phase are reinforced by the absolute differences in probabilities at 21 days from admission, shown in Table 2.

**Table 2:** Estimated impact of the intervention on the three outcomes using hazard ratios (relative) and the difference in probability at 21 days (absolute). The cells show the mean and 95% confidence interval

Outcome	Measure	Hospital		
		X	Y	Z
Clinician-led care review discussion	Relative	0.96 (0.85 to 1.09)	0.54 (0.42 to 0.69)	1.17 (0.99 to 1.37)
	Absolute	−0.03 (− 0.07 to 0.01)	−0.20 (− 0.28 to − 0.13)	0.05 (0.00 to 0.10)
Review of care directive measures	Relative	0.79 (0.68 to 0.92)	0.57 (0.43 to 0.74)	1.11 (0.97 to 1.28)
	Absolute	−0.08 (− 0.12 to − 0.04)	−0.14 (− 0.21 to − 0.06)	0.04 (0.00 to 0.08)
Palliative care referral	Relative	1.35 (0.90 to 2.04)	0.92 (0.58 to 1.44)	1.24 (0.92 to 1.67)
	Absolute	0.02 (−0.01 to 0.06)	−0.01 (− 0.07 to 0.04)	0.04 (− 0.01 to 0.08)

There was a reduction in review of care directive measures during the intervention compared with usual care and reduced probability at 21 days in Hospital X and in Hospital Y. There was a large reduction in clinician-led care review discussions at 21 days in hospital Y. There was little change in palliative care referrals in any hospital.

The change in completed outcomes during the intervention phase is similarly reflected in the hazard ratios from the Cox proportional hazards models in Table 2. The hazard ratios and the associated confidence intervals are below one for review of care directives measures in Hospitals X and Y and for clinician-led care review discussion in Hospital Y, in all cases indicating a reduced hazard for patients experiencing the outcome in the intervention phase. The hazard ratios for the patient characteristics are in Additional file 3: Table S2. The hazard ratios excluding prior outcomes are in Additional file 3: Table S3, most results were similar although in Hospital Z there was an increase in care directives during the intervention after excluding prior outcomes.

Large fluctuations in at-risk patient numbers are seen over time in each hospital (Additional file 3: Fig. S3). After detailed discussions with Queensland Health staff, we believe these fluctuations to be associated with: increased admission and discharge activity over the Christmas and new year period; changes in hospital processes, including how patients are admitted to clinical teams from the emergency department at one hospital; the allocation of beds for COVID patients; as well as public hesitancy around presenting to hospital for non-emergency appointments due to the COVID-19 pandemic.

## Discussion

This trial examined older patients with median age of 84 years who were largely (60%) ‘at-risk of death’ to evaluate the impact of introducing a prospective feedback loop as a prompt for clinicians’ responses to prevent or reduce non-beneficial treatment. It was anticipated that the knowledge of patient risk would trigger a proactive response, with actions such as improving time to clinical review discussion, reducing time to documented care directive measures - such as completion of an acute resuscitation plan - or accelerating palliative care referrals. However, the intervention did not achieve the goal of prompting clinicians to respond to notifications of at-risk patients. The reasons for this failure may be a combination of the intervention design and health system challenges, which we discuss below.

We used two tools to identify patients near the end of life. Even though these are validated for predicting short-term deterioration and death in older people, both were unfamiliar to most hospital clinicians participating in the trial and as such may not have been the effective ‘flag’ that would prompt an appropriate action. Other effective ‘flags’ used in Australian hospitals include criteria for patients at risk of pressure areas [16], falls [17], and criteria for enacting rapid response to serious patient deterioration [18]. These are measured and the response protocolised as a part of routine care, not measured by a third person and used to increase clinician awareness as in the case of our intervention. Moreover, both tools used in the trial are complex, and time consuming to be implemented into clinical practice unless the flags and direct prompts are automated, clinician time remunerated, and pathways of care enabled within services.

During the intervention phase, a two-fold notification system was used to alert clinicians of their at-risk patients on screening days, Mondays and Thursdays. A continuous system may have been more effective as the timing of information is essential to inform decision making and care planning.

Each hospital used a different initial notification. In two hospitals, the initial notifications were embedded in the electronic patient record - these were not highly visible and therefore may have been ineffective. The second notification - an email at the end of each screening day - was consistent across all hospitals, however this was often received after treatment decisions had been made. A more visible and time relevant system to flag at-risk patients may have been more successful.

Changing clinician behaviour is complex, especially in an area as sensitive as end of life. Reasons for this complexity are varied, including prognostic uncertainty, clinicians’ inexperience with death and dying, their orientation towards cure and cultural beliefs [4, 19] as well as palliative care having a somewhat negative connotation for some clinicians [20].

Nudge interventions have been used to attempt to improve the delivery of health care and review patients’ treatment plans. Our nudge intervention of a feedback loop was inherently passive. In co-designing the trial intervention with clinical teams, consideration was required to ensure that the audit feedback loop was delivered in accordance with a clinician’s choice (email, flag, text, etc), did not undermine clinician autonomy, was not prescriptive, and did not interfere with routine clinical practice. As a result, the only trigger for clinician behaviour change or clinical response was the third-party flagging of at-risk patient status. The intervention therefore simply consisted of audit outcome feedback to treating clinicians, which either: raised awareness; raised awareness and triggered a clinical response; or could be overlooked or ignored. Furthermore, clinicians appear to have a higher response to nudges which ‘change default options’, or ‘enable choice’ as opposed to nudges which solely provide information through prompt reminders [21]. Passive and asynchronous nudge interventions, or those non-aligned with patient preferences have been found to be less successful than those that use an active, synchronous intervention that requires immediate action [22, 23].

In researching a response to identified at-risk patients, our trial assumed that medical staff, especially senior medical clinicians, are essential to changing the system, hence the notification to senior clinicians of their patients at risk. We evaluated the impact of medical actions, including documentation of goals of care, actions to achieve goals and referral to palliative care. While medically directed awareness and action may be important, it could be that constructing effective system change may be more effective if the patient and family are involved in change. Multi-component hospital interventions for older people’s care, including clinicians’ ownership of the care pathway strategies and stakeholder engagement in co-design, [24] are more resource intensive and time-consuming [25]. Their effectiveness is promising though is yet to be convincingly demonstrated [26]. Shared decision making that reflect the individuals’ attitudes, beliefs and preferences to articulate their own goals of care is essential to quality healthcare [27, 28], however these involve a complex and time-consuming process and therefore have not been widely used in acute care settings.

Another reason for the failure of our intervention may have been related to the trial being conducted during the COVID-19 pandemic. The study commenced in early 2020 coinciding with the initial COVID-19 wave in Australia and the initiation of social restrictions. COVID delayed the start of the trial, which meant that the change-over to the intervention phase moved closer to Christmas. We planned trial start times to avoid Christmas and January for phase change-overs due to senior staff leave and the annual movement of medical staff.

Both the control and intervention phases experienced hospital restrictions disrupting the operation of the trial. The COVID-19 suspension of the trial and subsequent hospital restrictions impacted the frequency and types of contact between trial staff, site study staff and participating clinicians. Meetings were delayed and changed to online conference calls and the trial coordinator was unable to attend the hospitals to assist and educate auditing staff during restricted times.

COVID-19 may have imposed competing priorities that diverted clinicians’ attention. Aged care facilities also changed practices during the pandemic with some periods where they sent far fewer of their residents to hospital due to fears around them contracting COVID in hospital. This may be a key reason for the substantial variation in at-risk patient numbers over time (Additional file 3: Fig. S3). When at-risk patient numbers were increased, this potentially created barriers to completing the three outcomes with a busier hospital and associated pressure to discharge patients leaving less time for sensitive issues to be addressed. Alternatively, there was more discussion among healthcare professionals and society in general about end-of-life care, particularly concerning older people, due to high rates of infection and deaths in that group [29]. This highlights a weakness in the stepped-wedge design as it is vulnerable to external influences over time confounding the intervention.

The results varied greatly between hospitals, with two hospitals showing negative results while the third was more positive. Therefore, we do not present an overall estimate of the intervention because it appeared to be hospital specific. It is not unusual for complex interventions to be context dependent [30] and we are investigating the translation aspects of the intervention in future work. The variable and contextual nature of the results in the three hospitals would make it difficult to recommend a standardised and scalable system across a large jurisdiction.

The failure to refer frail older people near the end of life for formal palliative care pathway is perhaps not surprising. The specialty of palliative care currently deals mainly with care of patients who are terminally ill with cancer and to a lesser extent with terminally ill patients with single organ failure such as renal failure, heart failure and respiratory failure. Due to low public awareness of the scope of palliative care, it is less common for this specialty to deal with the terminal stages of life for frail older people with non-cancer life-threatening conditions [31].

Further results will be reported once available. These may help to further understand the care received by older patients at the end of life and elucidate the detailed contextual and implementation factors that influenced how this intervention played out in practice.

## Supplementary Information

**Additional file 1: Supplementary file 1.** Screening tools.

**Additional file 2.** Additional statistical information.

**Additional file 3.** Supplementary Figures and tables.

## Data Availability

Synthetic versions of the datasets for this study are available in the GitHub repository, https://github.com/agbarnett/InterACT
