# Supplementary material for: Impact of a prospective feedback loop on care review activities in older patients at the end of life. A stepped-wedge randomised trial
Source: BMC Geriatr. 2022 Nov 16;22:860. doi: 10.1186/s12877-022-03554-x (PMC9666964; doi:10.1186/s12877-022-03554-x)
Supplement: Supplementary file 1 — Additional file 1: Supplementary file 1. Screening tools. [file 12877_2022_3554_MOESM1_ESM.docx]

**CriSTAL Tool**

| **Date: ___/___ /_____** | | | **Consultancy team:** |
| --- | --- | --- | --- |
|  | | | |
|  | **Age >75** *(1 point)*  Admitted via Emergency Department *(1 point)* | | |
|  | Nursing home resident /in supported accommodation *(either: 1 point)* | | |
|  | Meets >= selected deterioration criteria on admission *(max 1 point if it meets >2 RRT criteria)* | | |
|  |  | 1 - Decreased LOC: Glasgow Coma Score change >2 or AVPU =P or U | |
|  |  | 2 - Systolic blood pressure <90 mmHg | |
|  |  | 3 - Respiratory rate <5 or >30 per minute | |
|  |  | 4 - Pulse rate <40 or >140 per minute | |
|  |  | 5 - Need for oxygen therapy or known oxygen saturation <90% | |
|  |  | 6 - Hypoglycaemia: BGL ­1.0 - 4.0 mmol/L | |
|  |  | 7 - Repeat or prolonged seizures (> once in 24 hours or >5 minutes duration) | |
|  |  | 8 - Low urinary output (<15 ml/hour or <0.5 ml/kg/hour) | |
| **AND** | **OTHER RISK FACTORS /PREDICTORS** (*tick as many as relevant- max 7 points*) | | |
|  | Personal history of active disease: | | |
|  | 1 - Advanced malignancy (Stage III or IV for solid tumors, or, metastatic, terminal, incurable) | | |
|  | 2 - Chronic kidney disease (Stage IV or V or GFR < 30mL/min) | | |
|  | 3 - Chronic heart failure (NYHFC III or IV or heart failure with shortness of breath with activity (III) or at rest (IV)) | | |
|  | 4 - Chronic obstructive pulmonary disease (GOLD criteria 3 severe FEV1 30-40% or 4 very severe FEV1 <30%) | | |
|  | 5 - New cerebrovascular disease (New stroke as reason for hospital admission, or stroke during this admission) | | |
|  | 6 - Myocardial infarction, new or pre-existing (documented EGC changes / Troponin rise) | | |
|  | 7 - Moderate/severe liver disease (Cirrhosis stage III or IV or liver cancer) | | |
|  | Evidence of cognitive impairment *(tick as many as relevant – only 1 point if >1 mental condition)*  Long term mental disorder  Dementia  Behavioural alterations (acute or chronic, including confusion or delirium)  Mental disability from stroke | | |
|  | Proteinuria on a spot urine sample: ++ or >30 mg albumin/g creatinine  ☐ Yes *(1 point)* ☐ No ☐ unknown | | |
|  | Abnormal ECG (atrial fibrillation, ventricular tachycardia, other abnormal rhythm or >5 ectopics/min, changes to Q or ST waves) *(tick as many as relevant – only 1 point if >1 abnormality)*  Acute abnormality  Chronic abnormality  Both chronic and acute this assessment  No abnormality  Don’t know | | |
|  | **Previous hospitalisation for at least one night in past year** (*only 1 point if >1 hospital admission)*  Yes  No  Not documented  Total No. of hospitalisations in the past year ______  **Previous ICU admission for at least one night in past year** (*only 1 point if >1 ICU admission)*  Yes  No  Unknown | | |
| **AND** | **Evidence of frailty (Clinical Frailty score)** | | |
|  | Rockwood < 5 ☐ Yes *(0 point)* Rockwood 5 or 6 ☐ Yes *(1 point)* Rockwood ≥7 ☐ Yes *(2 points)*  Actual CFS score *______* | | |

CriSTAL score ______

**
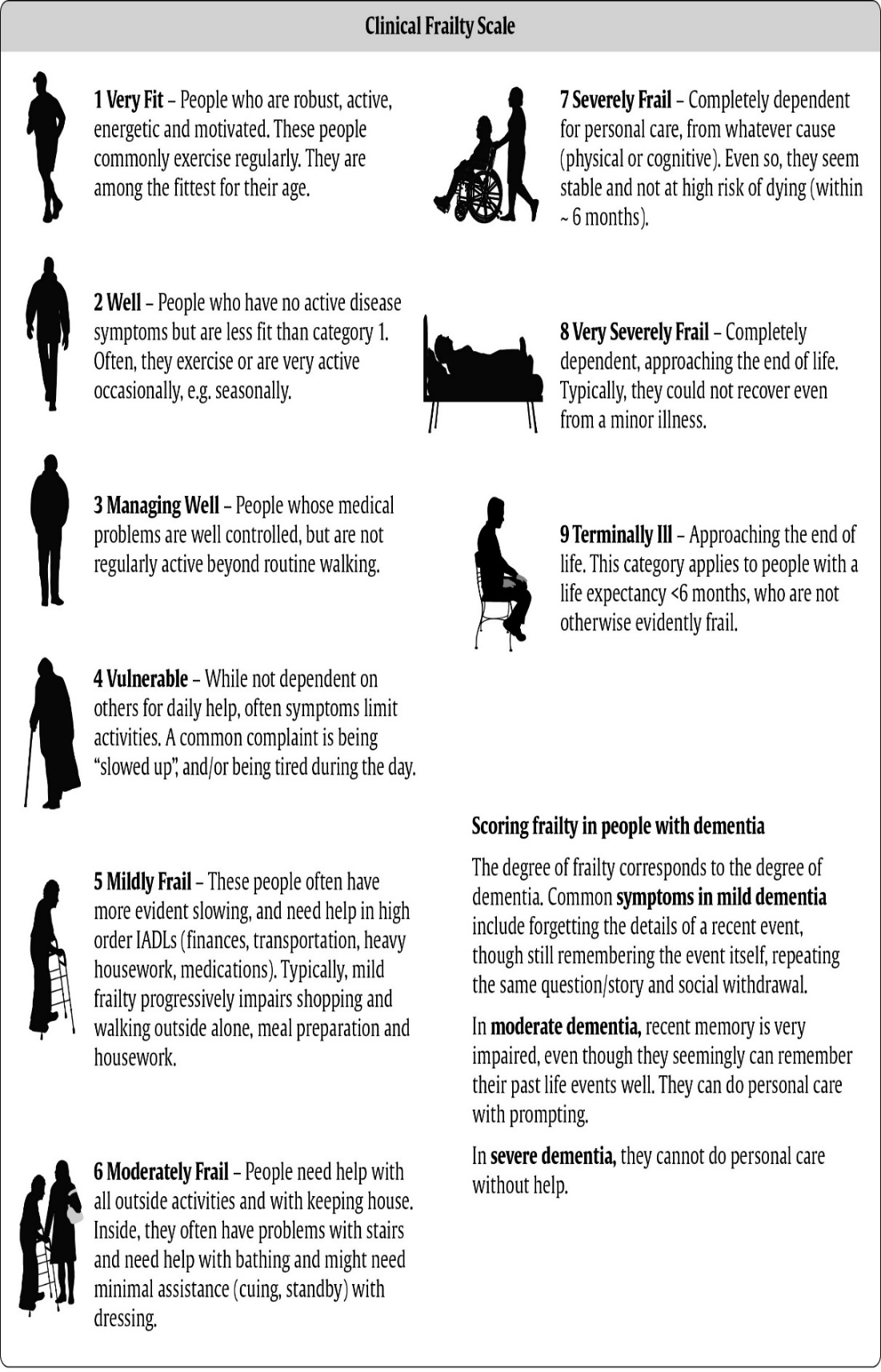
**

**Supportive and Palliative Care Indicators Tool (SPICT^TM^)**

InterACT screening only uses these initial six General Indicators of SPICT

| **The SPICT^TM^ is used to help identify people whose health is deteriorating.**  **Assess them for unmet supportive and palliative care needs. Plan care.** | | | |
| --- | --- | --- | --- |
| **Date: ___/___ /_____** | | | **Consultancy team:** |
| **Look for any general indicators of poor or deteriorating health.** | | | |
|  | Unplanned hospital admission(s) *(1 point)* | | |
|  | Performance status is poor or deteriorating, with limited reversibility.  (e.g. The person stays in bed or in a chair for more than half the day) *(1 point)* | | |
|  | Depends on others for care due to increasing physical and/or mental health problems. | | |
|  |  | The person’s carer needs more help and support. *(1 point)* | |
|  | Progressive weight loss; remains underweight; low muscle mass *(1 point)* | | |
|  | Persistent symptoms despite optimal treatment of underlying condition(s) *(1 point)* | | |
|  | The person (or family) asks for palliative care; chooses to reduce, stop or not have treatment; or wishes to focus on quality of life *(1 point)* | | |
| **Look for clinical indicators of one or multiple life-limiting conditions** | | | |
| **Cancer** | | | |
|  | Functional ability deteriorating a due to progressive cancer. | | |
|  | Too frail for cancer treatment or treatment is for symptom control. | | |
| **Dementia/frailty** | | | |
|  | Unable to dress, walk or eat without help. | | |
|  | Eating and drinking less; difficulty with swallowing. | | |
|  | Urinary and faecal incontinence. | | |
|  | Not able to communicate by speaking; little social interaction. | | |
|  | Frequent falls; fractured femur. | | |
|  | Recurrent febrile episodes or infections; aspiration pneumonia. | | |
| **Neurological disease** | | | |
|  | Progressive deterioration in physical and/or cognitive function despite optimal therapy. | | |
|  | Speech problems with increasing difficulty communicating and/or progressive difficulty with swallowing. | | |
|  | Recurrent aspiration pneumonia; breathless or respiratory failure. | | |
|  | Persistent paralysis after stroke with significant loss of function and ongoing disability. | | |
| **Heart/ vascular disease** | | | |
|  | Heart failure or extensive, untreatable coronary artery disease; with breathlessness or chest pain at rest or on minimal effort. | | |
|  | Severe, inoperable peripheral vascular disease. | | |
| **Respiratory disease** | | | |
|  | Severe, chronic lung disease; with breathlessness at rest or on minimal effort between exacerbations. | | |
|  | Persistent hypoxia needing long term oxygen therapy. | | |
|  | Has needed ventilation for respiratory failure or ventilation is contraindicated. | | |
| **Kidney disease** | | | |
|  | Stage 4 or 5 chronic kidney disease (eGFR <30ml/min) with deteriorating health. | | |
|  | Kidney failure complicating other life limiting conditions or treatments. | | |
|  | Stopping or not starting dialysis. | | |
| **Liver disease** | | | |
|  | Cirrhosis with one or more complications in the past year:   - Diuretic resistant ascites - Hepatic encephalopathy - Hepatorenal syndrome - Bacterial peritonitis - Recurrent variceal bleeds | | |
|  | Liver transplant is not possible | | |
| **Other conditions** | | | |
|  | Deteriorating and at risk of dying with other conditions or complications that are not reversible; any treatment available will have a poor outcome. | | |
