# Supplementary material for: Impact of a prospective feedback loop on care review activities in older patients at the end of life. A stepped-wedge randomised trial
Source: BMC Geriatr. 2022 Nov 16;22:860. doi: 10.1186/s12877-022-03554-x (PMC9666964; doi:10.1186/s12877-022-03554-x)
Supplement: Supplementary file 2 — Additional file 2. Additional statistical information. [file 12877_2022_3554_MOESM2_ESM.docx]

**Additional statistical information**

**Survival analysis**

Hospital discharge and death were treated as a combined competing event (Wolkewitz et al., 2014). For this paper, these were combined as we only collected the patients’ final date with the clinical team, i.e., their discharge from hospital or the clinical team, and not their final status. Data on patients’ final status will be made available to the study team at a future date and reported in a separate paper. This paper is concerned with outcomes other than death and discharge, therefore combining death and discharge is not a concern for this analysis, but there is a need to control for censoring from these competing events.

We used cumulative probability plots to illustrate the effect of the intervention on the three outcomes. These distinguish between informative censoring due to death and discharge which are competing risks, and censoring from patients not experiencing any event before their follow-up ended. The more familiar Kaplan–Meier plots cannot account for competing risks and will likely give biased estimates of survival (Gooley et al., 1999).

The sub-distribution model gives an estimate on an absolute scale. We also used a cause-specific hazards survival model to give relative estimates (hazard ratios), as recommended when reporting survival models (Kragh Andersen et al., 2021). The model used clinical teams as strata which meant that each team has its own baseline hazard which adjusted for differences between teams. The models’ residuals were assessed for patterns and outliers, and influential observations were checked using the df-beta statistic (Dobson & Barnett, 2018). There were a handful of patients with relatively high df-beta statistics and we investigated the characteristics of these patients to understand their influence, which often showed that they we patients with a high comorbidity, but who were discharged relatively early. These moderately influential patients had no impact on the overall results.

Both the Fine–Gray model and the Cox model adjusted for the patients’ age, sex, CriSTAL score and SPICT score. A binary variable indicated if the patient was part of the usual care or intervention phase. The four-week establishment phase was excluded from all models and the cumulative probability plots.

For the cumulative risk plots we show the differences up to 15 days, whereas for the survival models we model the difference at 21 days. This difference is because we wanted the plots to focus on when most events occur and better highlight any differences between groups. The difference in the model at 21 days captures almost all the events and gives a clinically meaningful time frame for judging the effect of the intervention. Showing the plots to 21 days would have given more consistent results, but would also have compressed some interesting changes over time during the first week.

We used a Cox model for the cause-specific hazards survival model and graphically checked the proportional hazard assumption (Supplementary figure A).

Supplementary figure A. Graphical check of the proportional hazard assumption. The grey circles are the residuals and the green line is the smoothed residuals with 95% confidence intervals denoted by the red shaded areas.

1. Clinician- led care review discussion


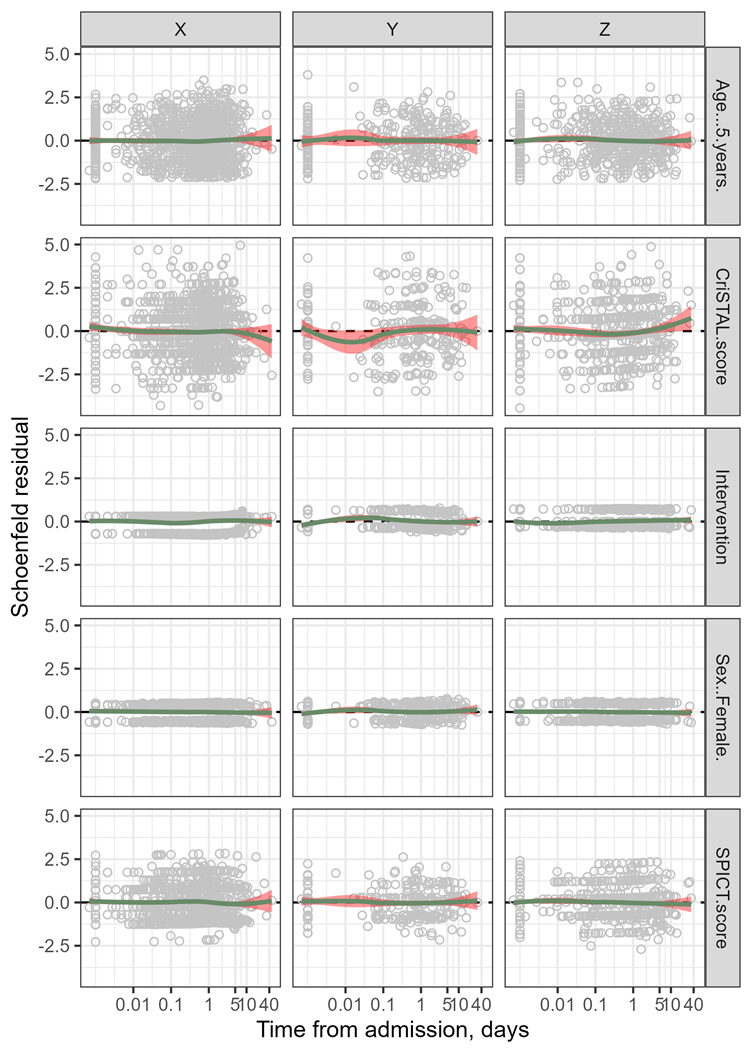


For clinical-led care review discussion, there is a small non-proportionality for CriSTAL in Hospital Y with a possibly weaker influence of CriSTAL at the start of the admission.

1. Review of care directive measures


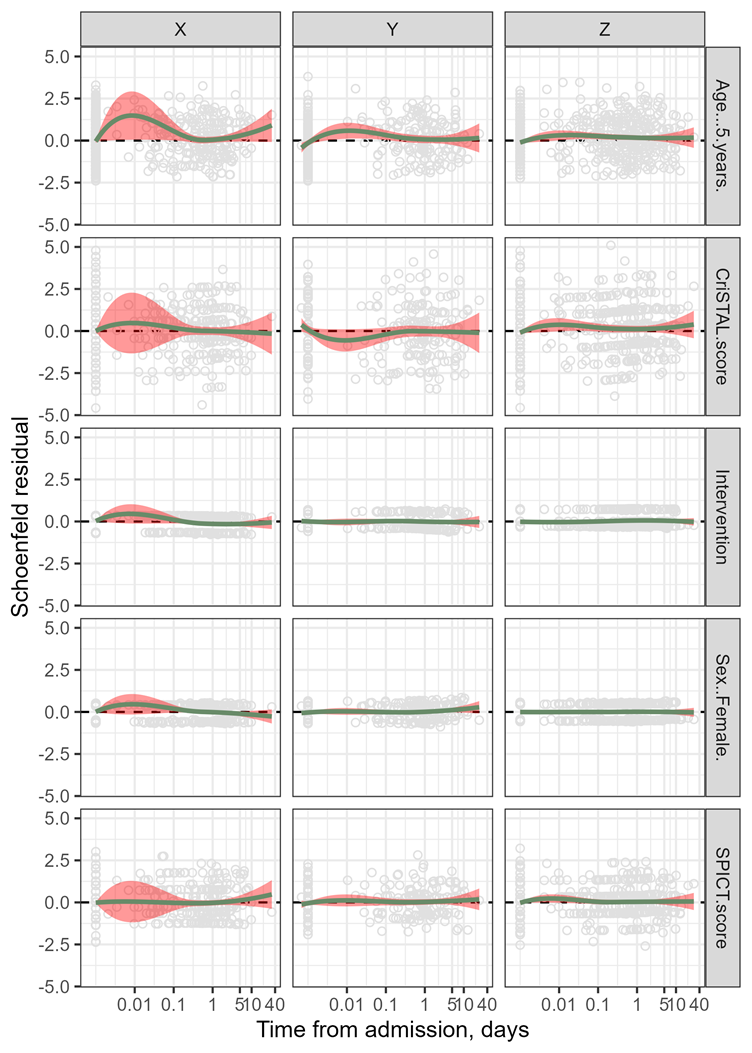


For review of care directive measures, there is some evidence of non-proportional hazards for age. An older age may have a stronger effect early in the admission.

1. Palliative care referral


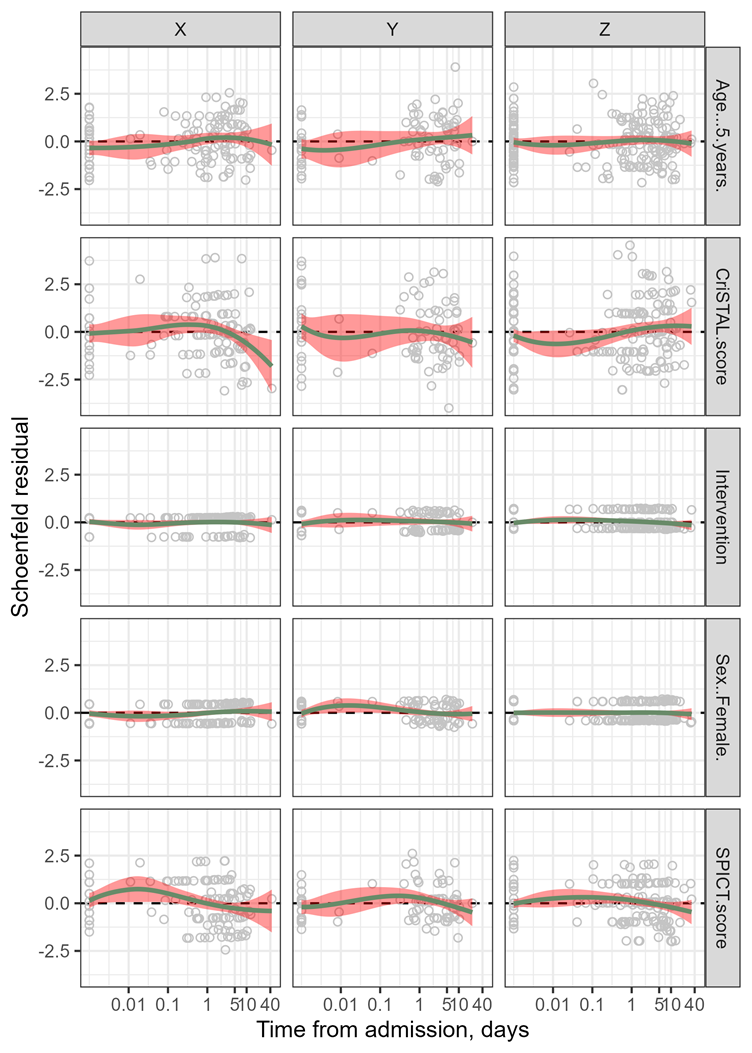


For palliative care referrals, SPICT score may have a stronger effect earlier in the stay. The effect of CriSTAL may be different for patients who have already spent many days in hospital.

**Intervention error**

One team at Hospital Z did not receive any emails during the intervention period due to a programming error in our automated system. This team therefore did not receive the intervention and so were analysed as part of the control group. This was one of the smaller teams and an analysis that included their intervention phase data as part of the intervention phase showed almost no difference in the hospital-level estimates of relative and absolute intervention effect at 21 days.

**Missing data**

There were some missing data for the review of care directives outcome, specifically, Acute Resuscitation Plans (ARP) and we used multiple imputation to account for this. See Supplementary figure B for a report on the amount of missing data using a multinominal model to impute the data.

Supplementary figure B. Missing data, ARP


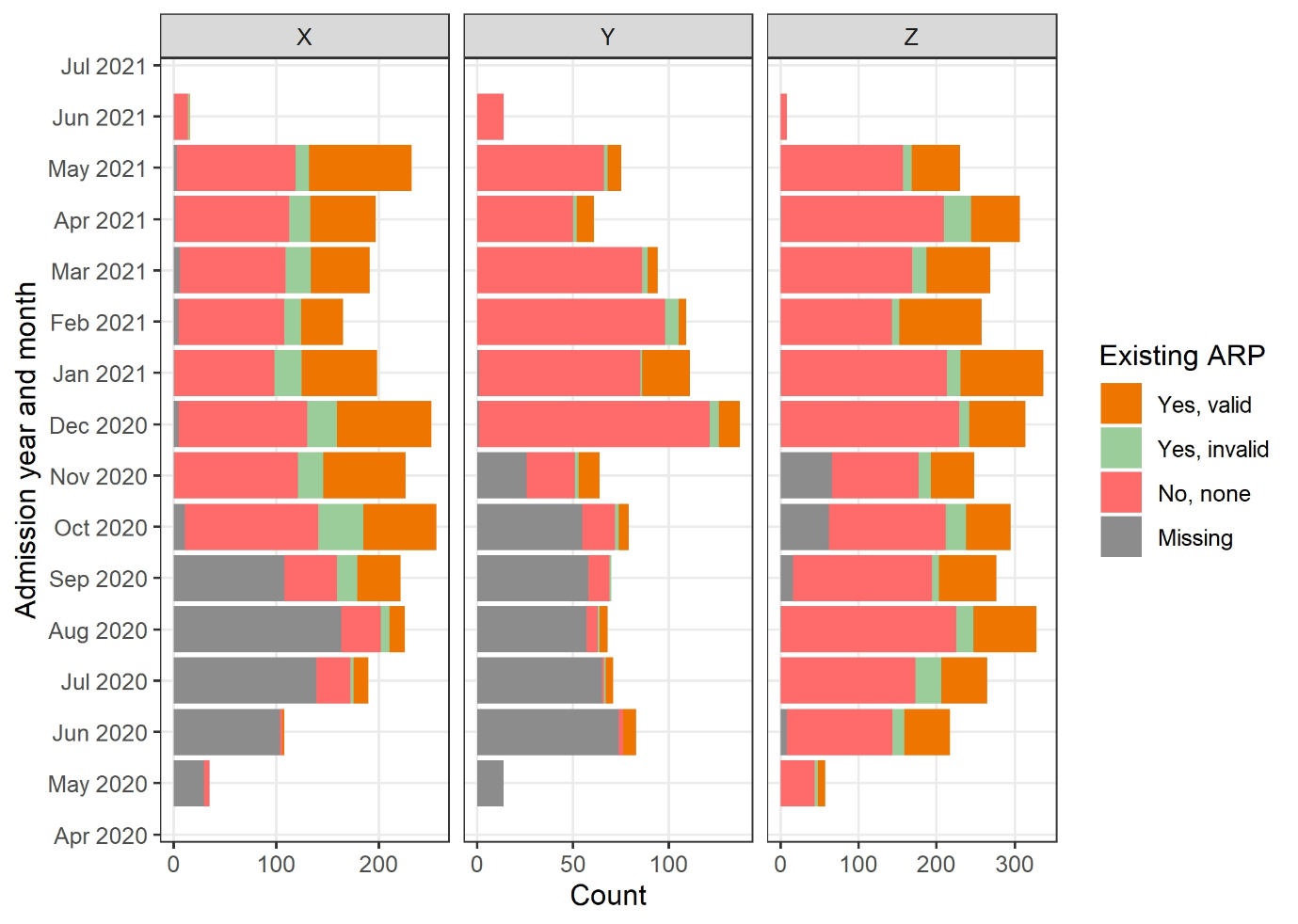


**Changes over time**

To investigate other changes over calendar time that might have impacted the outcomes, we plotted the final event for patients in narrow windows of time (Additional file 3, Figure 4). For each hospital and day of the study period *d* = 1,…,403, we used a window that included all patients in the next 30 days (*d*, *d*+30). We then plotted the final status for these patients, which was one of: prior outcome, positive outcome, discharged/died, or censored. The aim was to look for patterns over time caused by important events such as hospital lockdowns in reaction to COVID-19.

**Prior outcomes**

Some patients already had care directives in place, specifically an existing acute resuscitation plan, when they came under the care of the clinical team (hereby referred to as ‘prior’ outcomes). In this situation they could not experience the outcome during their admission. These patients were counted as a positive outcome with the time to outcome deemed just over zero, as this ensures these patients are included in the group who receive the outcome of interest. In a sensitivity analysis we re-ran the survival models excluding “prior” outcomes to examine outcomes in the subgroup with the potential to experience the outcome during their stay.

**Sample size**

The trial’s sample size was powered for the primary outcome of ICU admission and hence is not relevant for this paper. The ICU admission data are not yet available to the study team as these data are supplied to the statistics branch of the state’s health department and validated prior to provision to the study team.

**References**

Dobson, A. J., & Barnett, A. G. (2018). *An introduction to generalized linear models* (4th ed.). Chapman and Hall / CRC.

Gooley, T. A., Leisenring, W., Crowley, J., & Storer, B. E. (1999). Estimation of failure probabilities in the presence of competing risks: new representations of old estimators. *Statistics in Medicine, 18*(6), 695-706.

<https://doi.org/10.1002/(SICI)1097-0258(19990330)18:6>

Kragh Andersen, P., Pohar Perme, M., van Houwelingen, H. C., Cook, R. J., Joly, P., Martinussen, T., Taylor, J. M. G., Abrahamowicz, M., & Therneau, T. M. (2021). Analysis of time-to-event for observational studies: Guidance to the use of intensity models. *Statistics in Medicine, 40*(1), 185-211. https://doi.org/10.1002/sim.8757

Wolkewitz, M., Cooper, B. S., Bonten, M. J., Barnett, A. G., & Schumacher, M. (2014). Interpreting and comparing risks in the presence of competing events. *Bmj, 349*, g5060. <https://doi.org/10.1136/bmj.g5060>
