# Supplementary material for: Impact of a prospective feedback loop on care review activities in older patients at the end of life. A stepped-wedge randomised trial
Source: BMC Geriatr. 2022 Nov 16;22:860. doi: 10.1186/s12877-022-03554-x (PMC9666964; doi:10.1186/s12877-022-03554-x)

**Supplementary file 3: Figures and tables**

**Supplementary Figure 1**: Cumulative probability curves for the three outcomes (solid lines) and the competing risk of discharge/death (dotted lines) by blinded hospital. The x-axis is the time since the patient came under the care of the clinical team and ends at 15 days this focuses on the most events. The y-axis is the cumulative probability of the outcome or death/discharge. The upper limits on the y-axes vary by outcome. The shaded areas are 95% confidence intervals.


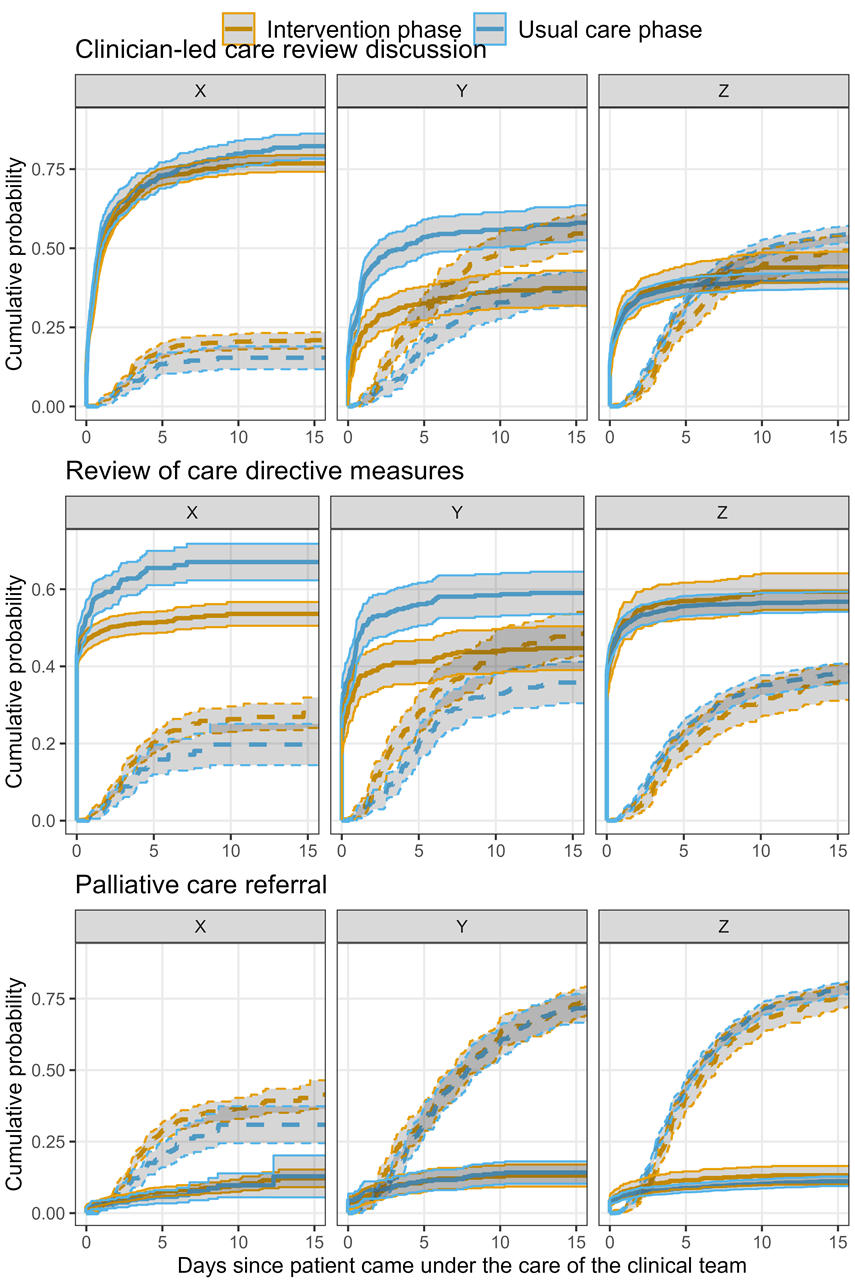


**Supplementary Table 1**: Numbers and percentages for the three outcomes by the two phases and three blinded hospitals. The “Yes” column shows the patients experiencing the outcome. “Censored” are patients whose admission ended before their outcome or discharge. “Discharge” include in-hospital deaths.

| Clinician-led care review discussion | | | | | | |
| --- | --- | --- | --- | --- | --- | --- |
| **Hospital** | **Phase** | **Censored** | **Discharge** | **Prior** | **Yes** | **Total** |
| X | Usual care | 74 (14) | 61 (12) | 44 (8) | 339 (65) | 518 (100) |
|  | Intervention | 174 (13) | 211 (16) | 122 (9) | 809 (61) | 1316 (100) |
| Y | Usual care | 3 (0) | 787 (60) | 159 (12) | 354 (27) | 1303 (100) |
|  | Intervention | 11 (2) | 253 (53) | 62 (13) | 154 (32) | 480 (100) |
| Z | Usual care | 0 (0) | 129 (42) | 48 (15) | 133 (43) | 310 (100) |
|  | Intervention | 5 (2) | 181 (61) | 12 (4) | 100 (34) | 298 (100) |
| Review of care directives measures | | | | | | |
| **Hospital** | **Phase** | **Censored** | **Discharge** | **Prior** | **Yes** | **Total** |
| X | Usual care | 132 (25) | 61 (12) | 221 (42) | 107 (21) | 521 (100) |
|  | Intervention | 428 (32) | 214 (16) | 534 (41) | 141 (11) | 1317 (100) |
| Y | Usual care | 3 (0) | 572 (44) | 481 (37) | 248 (19) | 1304 (100) |
|  | Intervention | 9 (2) | 179 (37) | 178 (37) | 114 (24) | 480 (100) |
| Z | Usual care | 0 (0) | 126 (41) | 90 (29) | 94 (30) | 310 (100) |
|  | Intervention | 3 (1) | 162 (54) | 65 (22) | 68 (23) | 298 (100) |
| Palliative Care Referral | | | | | | |
| **Hospital** | **Phase** | **Censored** | **Discharge** | **Prior** | **Yes** | **Total** |
| X | Usual care | 382 (77) | 87 (17) | 6 (1) | 24 (5) | 499 (100) |
|  | Intervention | 872 (68) | 318 (25) | 25 (2) | 73 (6) | 1288 (100) |
| Y | Usual care | 6 (0) | 1150 (88) | 54 (4) | 92 (7) | 1302 (100) |
|  | Intervention | 16 (3) | 400 (83) | 20 (4) | 44 (9) | 480 (100) |
| Z | Usual care | 0 (0) | 265 (85) | 13 (4) | 32 (10) | 310 (100) |
|  | Intervention | 9 (3) | 250 (84) | 9 (3) | 30 (10) | 298 (100) |

**Supplementary Figure 2**: Illustration of the survival data in one hospital for six hypothetical patients. There are three phases: usual care, establishment, and intervention. Patients are followed from their admission to the clinical team until they experience the outcome (O) or death/discharge (D). Patients in hospital at the start of the establishment phase or at the end of the study are censored.


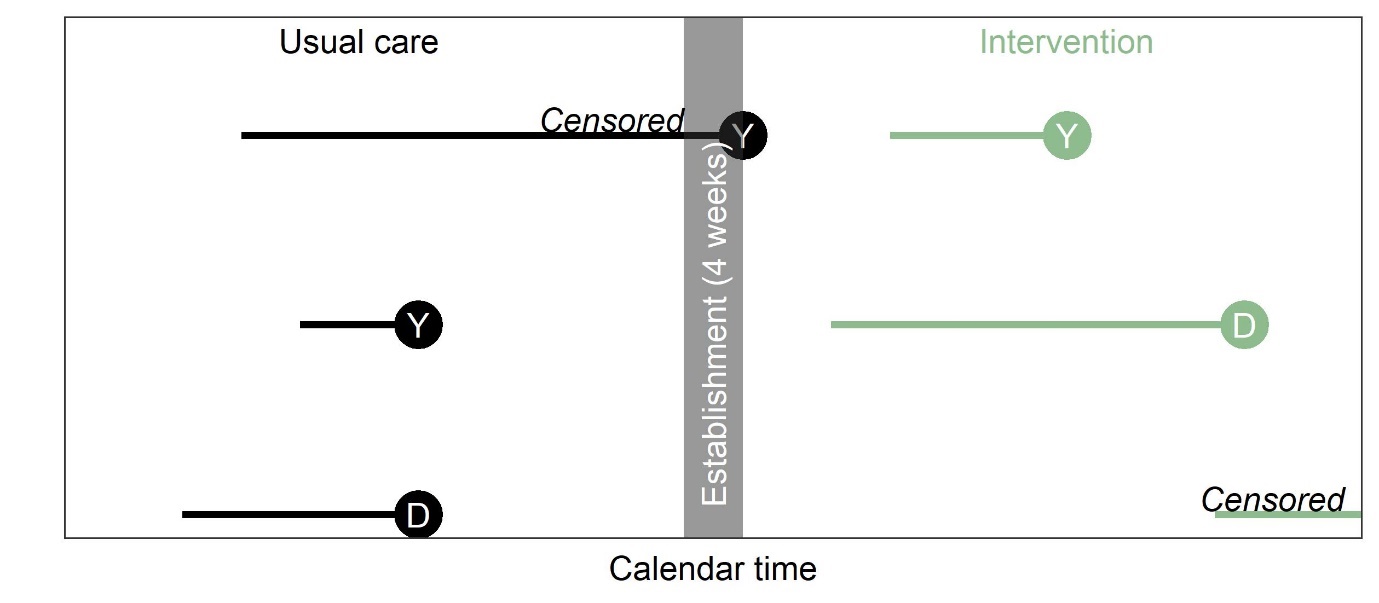


**Supplementary Table 2**: Hazard ratios and 95% confidence intervals for experiencing the three outcomes using Cox regression models. Results by blinded hospital.

| **Outcome** | **Variable** | **X** | **Y** | **Z** |
| --- | --- | --- | --- | --- |
| Clinical-led care review discussion | Age (+5 years) | 1.05 (1.01 to 1.10) | 1.23 (1.15 to 1.31) | 1.28 (1.16 to 1.42) |
|  | Sex (Female) | 0.94 (0.84 to 1.06) | 1.04 (0.90 to 1.20) | 1.21 (0.95 to 1.54) |
|  | CriSTAL score | 1.06 (1.02 to 1.10) | 1.31 (1.25 to 1.37) | 1.13 (1.05 to 1.21) |
|  | SPICT score | 1.09 (1.03 to 1.15) | 1.25 (1.16 to 1.34) | 1.29 (1.12 to 1.49) |
| Review of care directive measures | Age (+5 years) | 1.19 (1.12 to 1.27) | 1.20 (1.13 to 1.27) | 1.31 (1.18 to 1.45) |
|  | Sex (Female) | 0.91 (0.80 to 1.05) | 1.18 (1.04 to 1.34) | 0.97 (0.76 to 1.25) |
|  | CriSTAL score | 1.19 (1.14 to 1.25) | 1.24 (1.19 to 1.29) | 1.22 (1.13 to 1.31) |
|  | SPICT score | 1.14 (1.06 to 1.21) | 1.27 (1.19 to 1.36) | 1.14 (0.97 to 1.34) |
| Palliative care referral | Age (+5 years) | 0.94 (0.80 to 1.10) | 1.18 (1.04 to 1.34) | 1.20 (0.99 to 1.44) |
|  | Sex (Female) | 0.93 (0.65 to 1.33) | 0.59 (0.45 to 0.79) | 0.97 (0.62 to 1.51) |
|  | CriSTAL score | 1.46 (1.30 to 1.66) | 1.06 (0.97 to 1.15) | 1.38 (1.20 to 1.58) |
|  | SPICT score | 1.64 (1.38 to 1.96) | 1.60 (1.38 to 1.84) | 1.38 (1.06 to 1.80) |

**Supplementary Table 3:** Hazard ratios and 95% confidence intervals for experiencing ‘review of care directive measures’ outcome excluding prior outcomes, using Cox regression models. Results by blinded hospital.

| **Outcome** | **Variable** | **X** | **Y** | **Z** |
| --- | --- | --- | --- | --- |
| Review of care directive measures | Age (+5 years) | 1.35 (1.19 to 1.53) | 1.43 (1.24 to 1.65) | 1.43 (1.29 to 1.57) |
|  | Sex (Female) | 0.84 (0.64 to 1.11) | 1.20 (0.86 to 1.69) | 1.17 (0.95 to 1.45) |
|  | CriSTAL score | 1.16 (1.05 to 1.28) | 1.20 (1.09 to 1.33) | 1.30 (1.22 to 1.40) |
|  | SPICT score | 1.16 (1.00 to 1.34) | 1.18 (0.95 to 1.46) | 1.31 (1.17 to 1.47) |

**Supplementary figure 4:** At-risk patient numbers over calendar time and their categorical outcomes by blinded hospital. The dotted vertical line is the change-over time to the intervention. The scales on the y-axes vary by hospital.


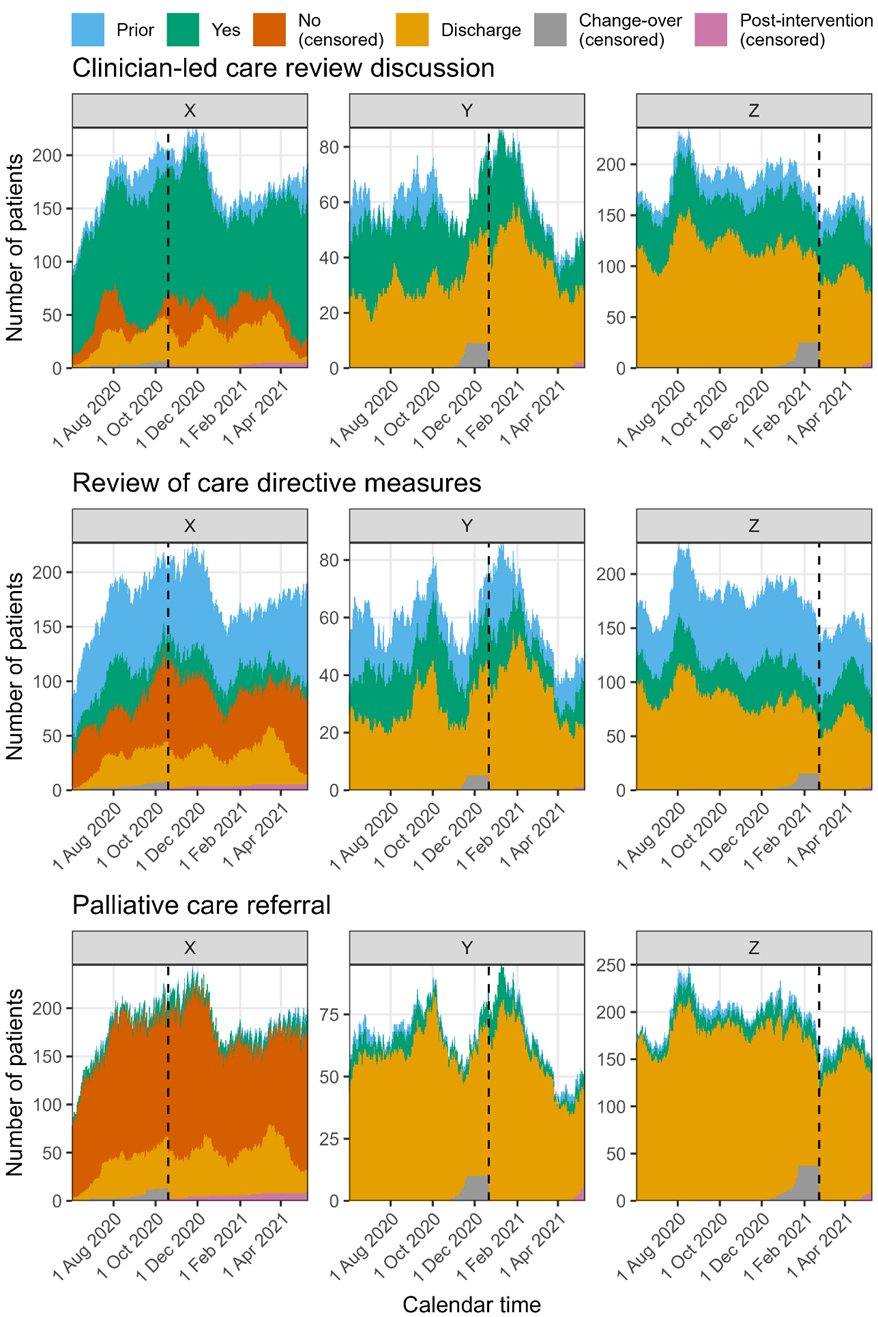

Supplement: Supplementary file 3 — Additional file 3. Supplementary Figures and tables. [file 12877_2022_3554_MOESM3_ESM.docx]
